# Multi-omics insights reveal the remodeling of gut mycobiome with *P. gingivalis*

**DOI:** 10.3389/fcimb.2022.937725

**Published:** 2022-08-29

**Authors:** Si Chen, ChenGuang Niu, WanQi Lv

**Affiliations:** ^1^ Department of Oral Implantology, Shanghai Stomatological Hospital and School of Stomatology, Fudan University, Shanghai, China; ^2^ Shanghai Key Laboratory of Craniomaxillofacial Development and Diseases, Fudan University, Shanghai, China; ^3^ Department of Endodontics, Shanghai Ninth People’s Hospital, Shanghai Jiao Tong University School of Medicine, College of Stomatology, Shanghai Jiao Tong University, Shanghai, China; ^4^ National Clinical Research Center for Oral Diseases, National Center for Stomatology, Shanghai Key Laboratory of Stomatology, Shanghai, China

**Keywords:** *porphyromonas gingivalis*, gut mycobiome, metagenomics, metabolomics, fungi-bacterial interaction, fungi-metabolite interaction

## Abstract

As a keystone periodontal pathogen, *Porphyromonas gingivalis* (*P. gingivalis*) was suggested to be involved in the progression of systemic diseases by altering the intestinal microecology. However, studies concerning gut microbiome have focused entirely on the bacterial component, while the fungal community (gut mycobiome) has been overlooked. In this study, we aimed to characterize the alteration of gut mycobiome profile with *P. gingivalis* administration using mice fecal samples. Metagenomic analysis showed a distinct composition pattern of mycobiome and significant difference of beta diversity between control and the *P. gingivalis* group. Some fungal species were differentially characterized with *P. gingivalis* administration, among which *Pyricularia pennisetigena* and *Alternaria alternata* showed positive correlation with *P. gingivalis*. KEGG functional analyses revealed that three pathways, namely, “pentose and glucuronate interconversions”, “metabolic pathways”, and “two-component system”, were statistically enriched with *P. gingivalis* administration. Moreover, the alteration of gut mycobiome was also closely related with serum metabolites, especially lipid and tryptophan metabolic pathways. Taken together, this study demonstrated the alteration of fungal composition and function with *P. gingivalis* administration for the first time, and investigated the fungi–bacterial interaction and fungi–metabolite interaction preliminarily, providing a whole insight into gut mycobiome remodeling with oral pathobiont through multi-omics analyses.

## Introduction

Periodontitis is the most common oral infection with a wide global prevalence and is characterized by the loss of tooth-supporting tissues (Kinane et al., 2017). The dysbiosis of oral microbial communities has been considered as the main cause of this disease (Curtis et al., 2020). *P. gingivalis*, a Gram-negative anaerobic bacterium, has been strongly implicated as a pathogen in the development of periodontitis. The detection rate of *P. gingivalis* in the periodontitis population ranges from 79% to 90%, which only accounts for 10% to 25% in the healthy population (Igboin et al., 2009). Moreover, the detrimental effects of *P. gingivalis* are not confined to the oral cavity; they can also contribute to systemic disorders, such as type 2 diabetes mellitus, cardiovascular disease, rheumatoid arthritis, and inflammatory bowel disease (IBD) (Hajishengallis and Chavakis, 2021).

The intestine harbors a complex diversity of microorganisms, consisting of bacteria, fungi, viruses, protozoa, and archaea, termed the gut microbiota. Due to the multiple functions revealed in the last decade, the gut microbiota has been recognized as a central regulator of some systemic disorders. Deciphering the mechanisms of host–intestinal microbiota interactions has been defined as a promising therapeutic strategy (Augs et al., 2021). The oral cavity and intestinal tract are linked by a constant flow of ingested food and saliva along the gastrointestinal tract, providing an opportunity for the translocation and colonization of oral microbiota (du Teil Espina et al., 2019; Schmidt et al., 2019). The oral–gut translocation of *P. gingivalis* has been proven in some gastrointestinal diseases, including IBD, colorectal cancer, nonalcoholic fatty liver disease, and other intestinal diseases (Komiya et al., 2019; Park et al., 2021). Recent studies have investigated the alterations of gut microbiota with *P. gingivalis* administration, though most of them are merely concerned about the bacterial microbiota (Nakajima et al., 2015; Ohtsu et al., 2019); the characteristics of other important components of the gut microbiome, such as fungi, remain undefined.

Fungi are an indispensable part of the intestinal microbiome and play a vital role in multiple physiological processes. Shotgun metagenomics suggests that fungi constitute ~0.1% of the human gut microbiome, which is termed the gut mycobiome (Huffnagle and Noverr, 2013). Research using germ-free mice indicated that the gut mycobiome could promote significant alterations in bacterial microbiome ecology and participate in the development of the innate and adaptive immune systems (Van Tilburg et al., 2020). Dysbiosis of the gut mycobiome has been illustrated in some systemic diseases, such as obesity, alcoholic liver disease, IBD, and COVID-19, by regulating the immune system and intestinal permeability (Sokol et al., 2017; Yang et al., 2017; Zuo et al., 2020). Moreover, the interaction between bacteria and fungi shapes the complex ecosystem and maintains intestinal homeostasis. For example, multiomics research based on 1,244 adults implicated that the gut mycobiome is interdependent on bacterial taxonomy and function, in which *Saccharomycetales* spp. interact with gut bacterial diversity to influence insulin resistance. Meanwhile, bacterial function participates in the effects of *Pichia* on blood cholesterol (Shuai et al., 2022).

Despite the emerging evidence of the importance of the gut mycobiome and fungi–bacterial interactions in health and disease, little research has focused on the remodeling of gut mycobiome with *P. gingivalis*. Our previous research demonstrated alterations in the gut bacteriome and serum metabolite profile induced by *P. gingivalis* administration (Dong et al., 2022). In this study, we aimed to demonstrate the alteration of the gut mycobiome with *P. gingivalis* administration, investigate the interaction between the gut mycobiome and bacteriome, especially *P. gingivalis*, and further explore the correlation of the gut mycobiome and serum metabolites preliminarily.

## Methods

### *P. gingivalis* cultivation

*P. gingivalis* strain ATCC33277 was obtained from ATCC, cultured in a brain–heart infusion (BD Bioscience, Franklin Lakes, NJ) consisting of 0.5% yeast extract (BD Bioscience), 10 mg/L hemin (Wako Chemicals, Osaka, Japan), and 1 mg/L 2-methyl-1,4-naphthoquinone (Tokyokasei, Tokyo, Japan) and incubated under anaerobic conditions (80% N_2_, 10% CO_2_, and 10% H_2_) at 37°C. The bacterial suspensions were prepared in phosphate-buffered saline (PBS) without Mg^2+^/Ca^2+^, and the optical density (OD) was measured at 600 nm with a standard curve.

### Animal experiments

C57BL/6 male mice at 8 weeks of age were obtained from Vital River Laboratory Animal Technology Company (Beijing, China) and group-housed in a specific pathogen-free (SPF) controlled environment with free access to food and water under a strict 12-h light/dark cycle. All animal experiments were approved by the Committee for the Care and Use of Laboratory Animals at Fudan University (Approval number: 202202006S). Sixteen mice were randomly divided into two equal groups. Mice in the *P. gingivalis* group were orally administered with 10^9^ CFU *P. gingivalis* twice a week for 6 weeks, and mice in the control group were administered PBS as a control.

### Sample collection

All animals were euthanized with carbon dioxide 6 weeks later. The colon contents were collected for metagenomic sequencing at Majorbio Bio-Pharm Technology Co. Ltd. (Shanghai, China). The serum samples were collected for untargeted metabolomics at Majorbio. The detailed information was demonstrated in our previous report (Dong et al., 2022).

### Untargeted metabolomics profiling

Untargeted metabolomics profiling of serum samples in the *P. gingivalis* and control groups was performed by Majorbio Bio-Pharm Technology Co. Ltd. (Shanghai, China). Chromatographic separation of the metabolites was performed on a Thermo UHPLC system equipped with an ACQUITY UPLC HSS T3 (100 mm × 2.1 mm i.d., 1.8 µm; Waters, Milford, USA). Mass spectrometry (MS) was performed using a Thermo UHPLC-Q Exactive Mass Spectrometer equipped with an electrospray ionization source operating in either positive or negative ion mode. Data acquisition was performed with the data-dependent acquisition mode. The detection was carried out over a mass range of 70–1,050 m/z. Raw data were imported into Progenesis QI 2.3 for peak detection and alignment. The preprocessing results contained the m/z values and peak intensity. The mass spectra of these metabolic features were identified using accurate masses. And the metabolites were searched and identified, and the main database was the HMDB (http://www.hmdb.ca/), Metlin (https://metlin.scripps.edu/) and Majorbio Database. The variable importance of the projection (VIP) score generated from orthogonal partial least squares discriminate analysis was used to determine the most differentiated metabolites. Metabolites with VIP ≥ 1.0 and *p*-value ≤ 0.05 were defined as significantly changed metabolites. A multivariate statistical analysis was performed using the R package ropls version 1.6.2.

### Metagenome taxonomic classification of fungal genomic reads

The DNA extract was fragmented to an average size of approximately 300 bp using Covaris M220 (Gene Company Limited, China) for paired-end library construction. Paired-end sequencing was performed on an Illumina NovaSeq/HiSeq Xten (Illumina Inc., San Diego, CA, USA) at Majorbio Bio-Pharm Technology Co., Ltd. (Shanghai, China) using NovaSeq Reagent Kits/HiSeq X Reagent Kits according to the manufacturer’s instructions (www.illumina.com). Reads were aligned to the mouse genome by BWA (http://bio-bwa.sourceforge.net), and any hits associated with the reads and their mates were removed. All predicted genes with 95% sequence identity (90% coverage) were clustered using CD-HIT (http://www.bioinformatics.org/cd-hit/), and the longest sequences from each cluster were selected as representative sequences to construct a nonredundant gene catalog. After quality control, reads were mapped to the representative sequences with 95% identity using SOAPaligner (http://soap.genomics.org.cn/), and the gene abundance in each sample was evaluated. Read counts assigned to the fungal kingdom were then extracted for analysis.

### Species and functional annotation

Representative sequences of the nonredundant gene catalog were aligned to the NCBI NR database with an e-value cutoff of 1e-5 using BLASTP (Version 2.2.28+, http://blast.ncbi.nlm.nih.gov/Blast.cgi) for taxonomic annotations. The Kyoto Encyclopedia of Genes and Genomes (KEGG) annotation was conducted using BLASTP (Version 2.2.28+) against the Kyoto Encyclopedia of Genes and Genomes database (http://www.genome.jp/keeg/) with an e-value cutoff of 1e-5.

### Supervised integration for interkingdom interactions

To identify the associations between the gut mycobiome and bacteriome or metabolome that were altered in the control and *P. gingivalis* groups, multi-omics datasets were integrated using the DIABLO framework from the mixOmics package in a supervised analysis. Standing for Data Integration Analysis for Biomarker discovery using Latent variable approaches for Omics studies, DIABLO is a novel multiomics framework for the integration of multiple datasets in a supervised analysis (Kong et al., 2022). A signature of 10 bacterial and 10 fungal species was selected for the integration of gut mycobiome and gut bacteriome. For the analysis of gut mycobiome and serum metabolome, the top 10 metabolites ranked with VIP (variable important in projection) value and the top 10 fungi by abundance were included. A design matrix of 0.1 was used to place higher importance on the discrimination of genotypes rather than maximizing the correlation between the two datasets. A signature of 10 bacterial and 10 fungal species was selected for the model. A correlation cutoff of 0.5 was set for the network visualization using Gephi.

### Statistical analysis

Alpha diversity was calculated using the Shannon index. The equality of variance was confirmed with Levene’s test. Normality of the data was evaluated using the Shapiro–Wilk test. The significance of genotype was assessed with the Student’s *t*-test.

Beta diversity was measured using the principal coordinate analysis (PCoA) with Bray–Curtis distance used to calculate the distance metric. Analysis of similarities (ANOSIM) test was used for the statistical analysis.

Linear discriminant analysis effect size (LEfSe) was used to identify the significant differential fungal biomarkers with a linear discriminant analysis (LDA) score greater than 3.5. The Kruskal–Wallis test was used to detect significant differences in abundance, and the Wilcoxon rank-sum test was used for *post-hoc* comparison. A *p*-value < 0.05 was considered significant.

For KEGG analysis, Wilcoxon rank-sum test was used for the differential pathways and KEGG orthology (KO), and FDR (false discovery rate) was performed on *p*-values. Two-tailed *p* < 0.05 was considered statistically significant. Correlation analysis was constructed to investigate the interaction between fungal species and KEGG pathways, and Spearman coefficient |*r*| > 0.5 and *p*-value < 0.05 are shown.

To investigate correlations between fungal taxa and Porphyromonadaceae or *P. gingivalis*, correlation networks with Spearman coefficient |*r*| > 0.6 and *p*-value < 0.05 were set. A heatmap was used to demonstrate the correlations between the 39 differential metabolites and the top 20 species in abundance. The Spearman coefficients represented by *R* values (range from −0.8 to 0.8) are shown in different colors. A *p*-value < 0.05 is marked with *, and a *p*-value < 0.01 is marked with **.

## Results

### Alterations in gut mycobiome composition with *P. gingivalis* administration

**Figure 1:**
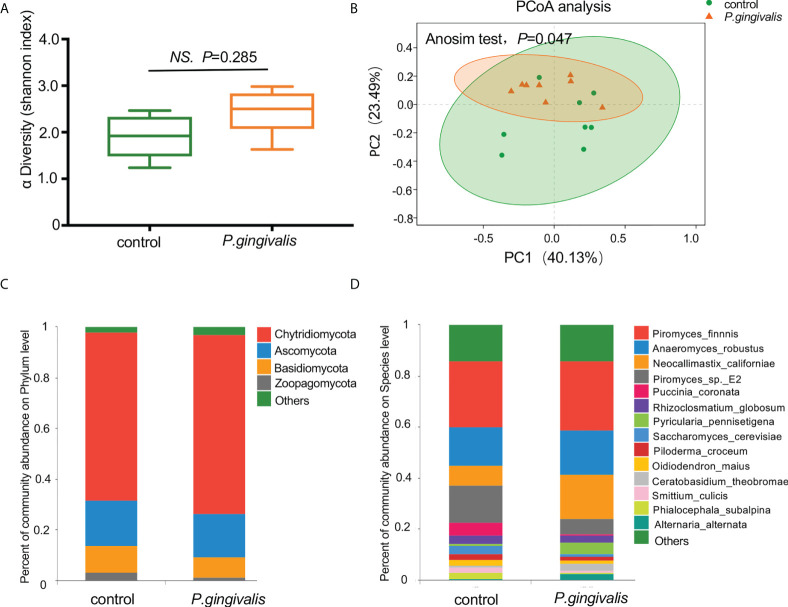
Diversity and composition of gut mycobiome with *P. gingivalis* administration in C57BL/6 mice. **(A)** Alpha diversity of fecal mycobiome measured by Shannon index between *P. gingivalis* and control mice (*t* test, *p* = 0.285). **(B)** Principal coordinate analysis (PCoA) plots of the gut mycobiome by Bray–Curtis distance between *P. gingivalis* and control mice (ANOSIM test, *p* = 0.047). **(C)** Mean relative abundances of gut fungi at the phylum level. **(D)** Mean relative abundances of gut fungi at the species level.

A total of 10,392 microorganisms were detected according to the metagenomic sequencing, among which 0.52% (54/10,392) of the reads aligned to fungal genomes. The alpha diversity analysis measured by the Shannon index demonstrated no significant differences in the richness of fungal communities with *P. gingivalis* administration (*t* test, *p* = 0.285, **Figure 1A**). The PCoA results measured by Bray–Curtis distance indicated a significant difference in the fungal diversity between the *P. gingivalis* and control groups (ANOSIM test, *p* = 0.047, **Figure 1B**). The composition of the gut mycobiome at different levels was then characterized. Chytridiomycota, Ascomycota, Basidiomycota, and Zoopagomycota constituted the main dominant phyla in *P. gingivalis*-treated mice, with mean relative abundances of 70.6%, 17.2%, 7.9%, and 1.4%, respectively. Mice in the control group demonstrated a different gut fungal pattern, consisting of 66.5% of Chytridiomycota, 17.7% of Ascomycota, 10.6% of Basidiomycota, and 3.2% of Zoopagomycota (**Figure 1C**). In particular, 54 kinds of fungi were detected at the species level (**Table S1**). *Piromyces finnis* was the most abundant species in both the *P. gingivalis* and control groups, with proportions of 27.1% and 25.9%, respectively, followed by *Anaeromyces robustus* (17.7%) and *Neocallimastix californiae* (17.1%) in the *P. gingivalis* group and *A. robustus* (15.1%) and *Piromyces_sp._E2* (14.7%) in the control group (**Figure 1D**).

### Identification of differential gut fungi with *P. gingivalis* administration

**Figure 2:**
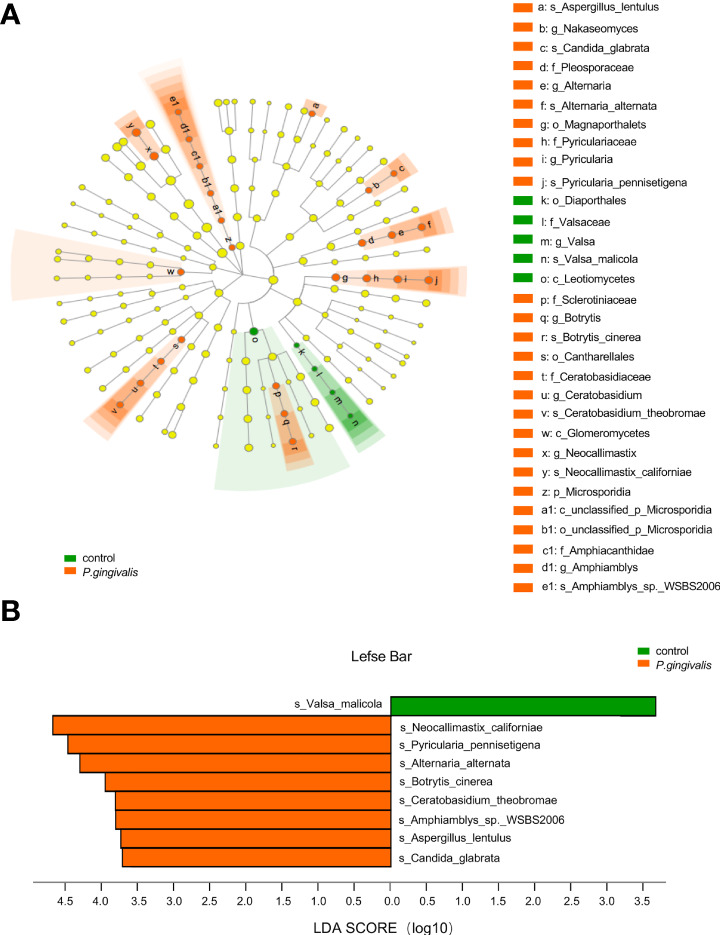
Differential gut fungi based on LEfSe between *P. gingivalis* and control groups. The differential fungal taxa are demonstrated based on an LDA score greater than 3.5 and marked by lowercase letters. **(A)** Cladogram of gut fungi at different levels. Each small circle at different taxonomic levels represents a taxon at that level, and the diameter of the circle is proportional to the relative abundance. Node in different color represents different group, and node in yellow represents no significant difference. **(B)** Significant altered gut fungi at the species level in the *P. gingivalis* and control groups. *p* < 0.05 with Wilcoxon rank-sum test followed by Kruskal–Wallis test.

To further identify the gut fungi with significant differences between the *P. gingivalis* and control groups, LEfSe was introduced. The differential taxa at various levels (phylum, class, order, family, genus, and species) based on an LDA score greater than 3.5 were identified (**Figure 2A**). Specifically, at the species level, *Valsa malicola* was abundant in the control group, while eight other species, namely, *N. californiae*, *Pyricularia pennisetigena*, *Alterbaria alternata*, *Botrytis cinerea*, *Candida glabrata*, *Aspergillus lentulus*, *Ceratobasidium theobromae*, and *Amphiamblys_sp_WSBS2006*, were abundant in the *P. gingivalis* group (**Figure 2B**). We further characterized the species with significant importance, and 20 of the top important species were listed with *P. gingivalis* administration according to the random forest algorithm (**Figure S1**). The most important species was *P. pennisetigena*, followed by *V. malicola* and *Amphiamblys_sp_WSBS2006*.

### Alterations in gut mycobiome function with *P. gingivalis* administration

**Figure 3:**
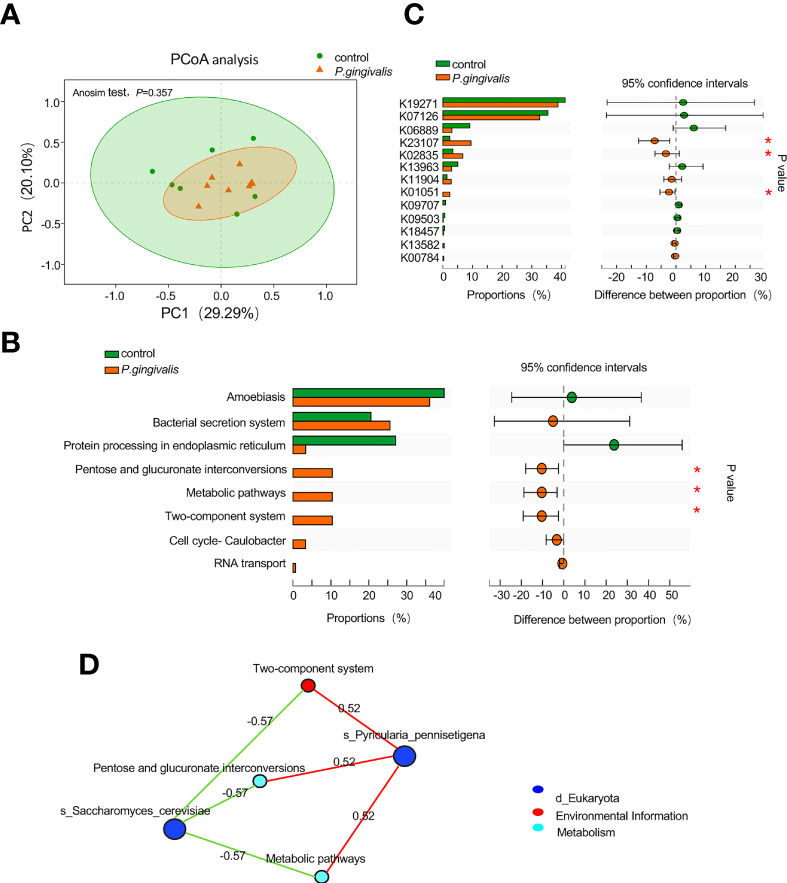
Functional alteration of gut mycobiome with *P. gingivalis* administration in C57BL/6 mice. **(A)** Principal coordinate analysis (PCoA) plots of the functional alterations by Bray–Curtis distance between *P. gingivalis* and control mice (ANOSIM test, *p* = 0.357). **(B)** Altered pathways in response to *P. gingivalis* administration based on the proportion of the abundance of each KEGG pathway. The 95% confidence intervals are presented with differences in the abundance of each pathway (Wilcoxon rank-sum test, *p < 0.05). **(C)** Altered KOs in response to *P. gingivalis* administration based on the proportion of KOs. The 95% confidence intervals are presented with differences in the KO abundance (Wilcoxon rank-sum test, *p < 0.05). **(D)** Correlation network analysis between functional alterations and differential fungal species. Spearman correlation of >0.5 or <− 0.5 was represented.

To investigate the potential biological function arising from alterations of the fungal community, the fungal genes were annotated to KO. The PCoA results based on Bray–Curtis distance showed no remarkable difference in the microbial function between the *P. gingivalis* and control groups (ANOSIM test, *p* = 0.357, **Figure 3A**). However, “pentose and glucuronate interconversions”, “metabolic pathways” and “two-component system” were statistically enriched with *P. gingivalis* administration (Wilcoxon rank-sum test, *p* < 0.05, **Figure 3B**). K23107, encoding 1-deoxy-D-xylulose 5-phosphate synthase, K02835, encoding peptide chain release factor 1, and K01051, encoding pectinesterase, were increased with *P. gingivalis* administration (Wilcoxon rank-sum test, *p* < 0.05, **Figure 3C**). Correlation network analysis between significant functional alterations and fungal species showed that *Pyriculari pennisetigena* had positive correlations with “metabolic pathways”, “pentose and glucuronate interconversions”, and “two-component system” (*r* = 0.52), while *Saccharomyces cerevisiae* demonstrated a negative correlation with the functional alteration (*r* = −0.57) (**Figure 3D**).

### Interactions between the gut mycobiome and bacterial microbiome

**Figure 4:**
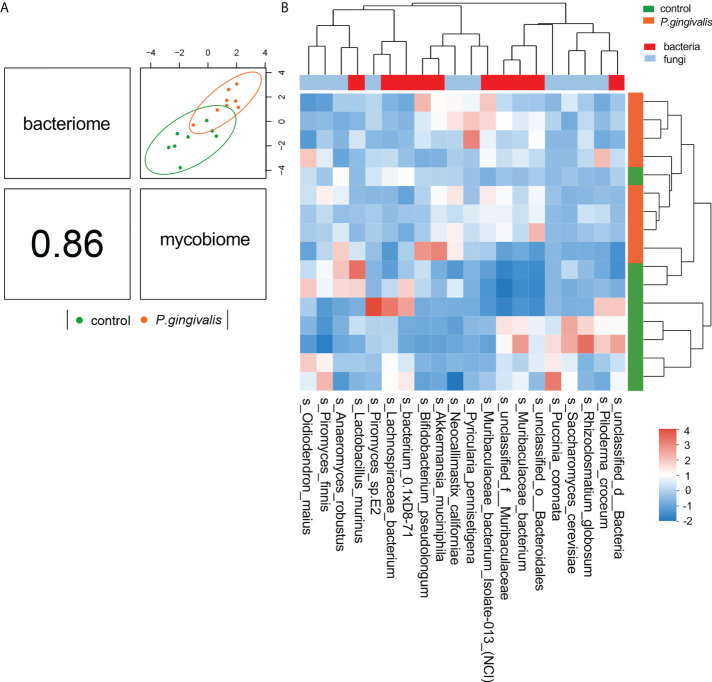
Integration of gut bacteriome and mycobiome. **(A)** The gut bacteriomes and mycobiomes were highly concordant with each other (*r* = 0.86) and revealed distinct clustering of samples from control and *P. gingivalis* mice. DIABLO was used for integration of gut bacteriome and mycobiome compositions. **(B)** Clustered heatmap of the abundances of bacterial and fungal species that covary with each other as shown in the column.

**Figure 5:**
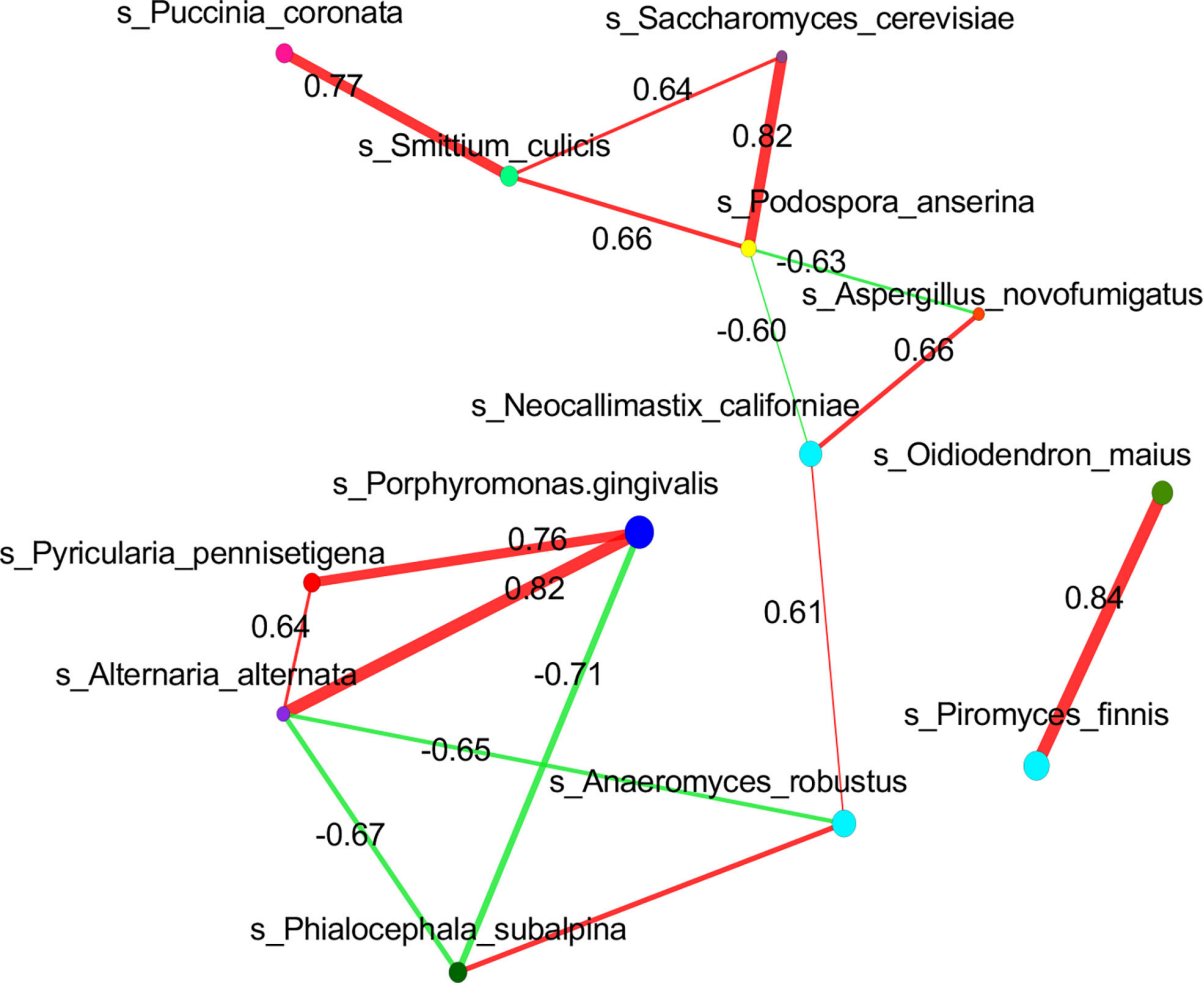
Correlation of *P. gingivalis an*d gut mycobiome at the species level. Correlation network analysis revealed strong positive and negative correlation associations between *P. gingivalis* and fungal species taxa. Spearman correlation of >0.6 or <−0.6 between fungal species was represented.

To investigate the relationship between mycobiome and bacteriome in the gut, we performed correlation analysis with the highest 10 species in abundance from the bacterial and fungal communities using DIABLO. With a design matrix of 0.1, which was set to maximize the discrimination between the two genotypes, the correlation between the gut fungal dataset and the gut bacterial dataset was still strong (*r* = 0.86) (**Figure 4A**). Components involved in the analysis were clustered as shown in **Figure 4B**. Additionally, correlation analysis was conducted between the abundance of *P. gingivalis* and the fungal mycobiome, and the Spearman coefficient was used to evaluate the correlation. At the family level, Porphyromonadaceae demonstrated a positive correlation with many fungal families, among which Pleosporaceae, Sclerotiniaceae, Pyriculariaceae, and Ceratobasidiaceae all showed positive correlations (*r* > 0.6) (**Figure S2**); these fungi are all recognized as pathogens and play important roles in the occurrence and progression of systematic diseases (Mahmoud et al., 2012; Klaubauf et al., 2014; Teifoori et al., 2018; Feng et al., 2021). In particular, two fungal species, *Alternaria alternata* and *P. pennisetigena*, possessed strong positive correlations with *P. gingivalis* (*r* = 0.82 and *r* = 0.76, respectively), while *Phialocephala subalpina* possessed a negative correlation (*r* = −0.71) (**Figure 5**).

### Correlation between the gut mycobiome and the serum metabolome

**Figure 6:**
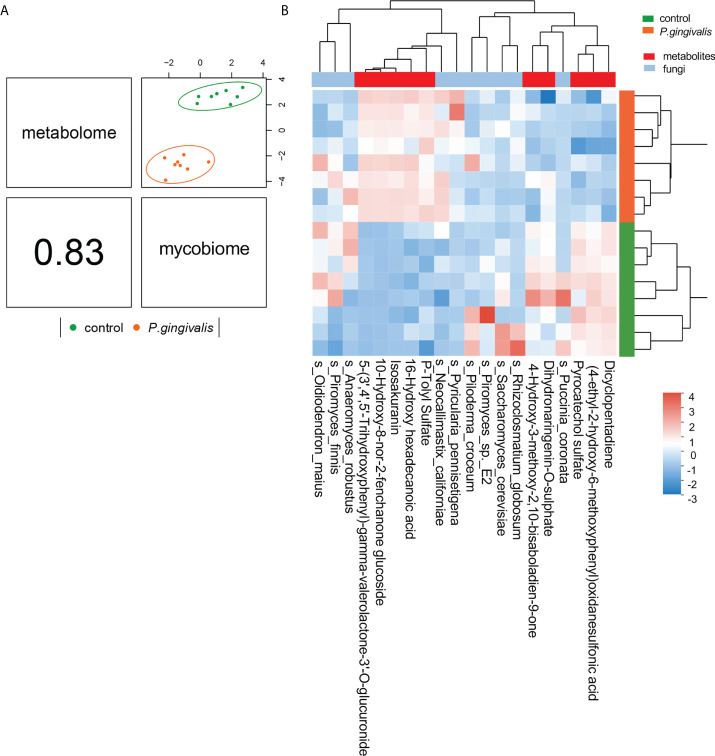
Integration of serum metabolome and gut mycobiome. **(A)** The serum metabolome and gut mycobiome were highly concordant with each other (*r* = 0.83) and revealed distinct clustering of samples from control and *P. gingivalis* mice. DIABLO was used for integration of serum metabolome and gut mycobiome compositions. **(B)** Clustered heatmap of the abundances of serum metabolites and fungal species that covary with each other as shown in the column.

Microbial metabolites are key factors in host–microbiota crosstalk. Our previous analysis confirmed 39 metabolism-related metabolites with *P. gingivalis* administration with untargeted metabolomics profiling (Dong et al., 2022). To further identify correlations between the fungi species and metabolic features that differed between the two groups, we integrated the two datasets using supervised analysis, and a strong correlation was concluded (*r* = 0.83) (**Figure 6A**). The top 10 metabolites by VIP value and the top 10 fungi by abundance were clustered for the analysis (**Figure 6B**). Correlations between the significantly altered metabolites and the gut mycobiome were further investigated, and *R* values of Pearson correlation coefficients and *P* values of significance were used to evaluate the correlations. A total of 20 gut fungi were identified with significant correlations. Notably, *Amphiamblys*, *P. pennisetigena*, and *V. malicola* were significantly correlated with most metabolites and were the top three most important species in the random forest ranking. As a more abundant species with *P. gingivalis* administration, *P. pennisetigena* was positively correlated with lipid metabolism-related metabolites, such as LysoPC, and negatively correlated with indole-3-acetamide, 5-hydroxy-tryptophan, and indoleacetaldehyde (**Figure S3**). All three metabolites are key actors in tryptophan metabolism, suggesting an interpretation of the gut mycobiome and functional synergism.

## Discussion

The current study demonstrates the first evidence of gut fungal dysbiosis with *P. gingivalis* administration. *P. gingivalis* alters the gut fungal composition, which correlates with metabolic pathways and serum metabolites, indicating an interactive relationship between the gut bacteriome and the gut mycobiome. Eight species were identified in the fecal samples of *P. gingivalis*-treated mice, among which *P. pennisetigena* ranked first due to its discrepant contribution and correlation with metabolic pathways.

The gut mycobiome accounts for less than 1% of the entire microbiome and has long been considered to be less important than the bacteriome. However, recent studies have confirmed the gut mycobiome as an integral part of the gut ecosystem (Hillman et al., 2017). As a periodontopathic bacterium, *P. gingivalis* plays a key role in the pathogenesis of periodontitis-associated comorbidities. In this study, a remarkable difference was observed in the fungal composition with *P. gingivalis* administration. In particular, *N. californiae*, *P. pennisetigena*, *A. alternata*, *B. cinerea*, *C. glabrata*, *A. lentulus*, and *C. theobromae* were significantly enriched in *P. gingivalis*-treated mice, and most of these fungi have been reported as pathogens in previous research. For example, *A. alternata* is a dematiaceous fungus and can cause cutaneous, even fatal, infection in humans (Sinha et al., 2021). *C. glabrata* can suppress immune responses and adapt to changing environmental conditions through a distinct set of virulence attributes (Bolotin-Fukuhara and Fairhead, 2014; Rasheed et al., 2020). *A. lentulus* is a drug-resistant species and has been identified as a main cause of azole-breakthrough disease (Nematollahi et al., 2021). *C. theobromae* is reported to be the causal agent of vascular streak dieback of cacao (Ali et al., 2019). *N. californiae* has been regarded as a member of the anaerobic gut fungi whose character has not been extensively explored but showed a positive correlation with total volatile fatty acids, including acetate, butyrate, and propionate (Wu et al., 2021).

Bacteria and fungi are in a dynamic balance. Some studies have demonstrated that disturbance of bacterial communities using long-term antibiotic treatments is often associated with fungal overgrowth and is ultimately linked to infectious diseases (Mason et al., 2012; Dollive et al., 2013). Sovran et al. revealed that the detrimental effects of *Candida albicans* and the beneficial effects of *Saccharomyces boulardii* in colitis depend on a specific bacterial environment of the Enterobacteriaceae population within the gut microbiota (Sovran et al., 2018). It has also been shown that commensal anaerobic bacteria—specifically Clostridial, Firmicutes, and Bacteroidetes—are critical in promoting *C. albicans* colonization resistance in mice by activating gut mucosal immune effectors (Fan et al., 2015). In our study, we found that administration of *P. gingivalis* not only changed the composition of intestinal bacteria but also altered the composition and function of intestinal fungi and further showed strong correlations with some gut fungi, illustrating the interaction between bacteria and fungi in health and disease.

Although “pentose and glucuronate interconversions”, “metabolic pathways”, and “two-component system” were statistically enriched with *P. gingivalis* administration though fungal KEGG annotations, the beta diversity of gut fungal function demonstrated no significant difference between the *P. gingivalis* and control groups, suggesting the potential role of the gut bacteriome. However, co-abundance network analysis revealed the mutual relationships between gut fungi and bacteria. In this study, *A. alternata* and *P. pennisetigena* possessed strong positive associations with *P. gingivalis*, while *P. subalpina* possessed a negative association. Broad associations between the gut mycobiome and the gut bacteriome and intestinal metabolites have been illustrated in recent studies (Shah et al., 2021; Shuai et al., 2022). Our previous research concerning the function of gut bacteria revealed that the “tryptophan metabolism pathway” was significantly altered with *P. gingivalis* administration and that indole and its derivatives were downregulated (Dong et al., 2022). Here, *P. pennisetigena* was shown to positively correlate with lipid metabolism-related metabolites, such as LysoPC, and negatively correlate with indole-3-acetamide, 5-hydroxy-tryptophan, and indoleacetaldehyde, which might be a potential reason for its correlation with the metabolic pathway. Acting as a pathogen for many plants, *P. pennisetigena* was found to be enriched in tuberculosis patients with bacteriologically confirmed infection compared with the negative group (Ding et al., 2021). In conclusion, it is reasonable to speculate that the systemic diseases associated with *P. gingivalis* or periodontitis may be related to the intestinal fungal alterations caused by *P. gingivalis*.

There are some limitations in our study. First, due to the technical difficulty of fungal culture, the correlations between gut fungi and the bacteriome and serum metabolites could not be validated *in vitro*. Moreover, we failed to relate these correlations between *P. gingivalis* and fungi to the phenotype. Many of the fungal species reported are of environmental origin, and their biological relevance in mammals remains unknown. Despite these findings, our study illustrated the remodeling of the gut mycobiome with *P. gingivalis* and further suggested the potential interrelationship between the gut mycobiome and metabolic functions and metabolites.

## Data availability statement

The datasets presented in this study can be found in online repositories. The names of the repository/repositories and accession number(s) can be found below: https://www.ncbi.nlm.nih.gov/sra/, PRJNA811648. https://www.ebi.ac.uk/metabolights/, MTBLS4106”.

## Ethics statement

This study was reviewed and approved by All animal experiments were approved by the Committee for the Care and Use of Laboratory Animals at Fudan University (Approval number: 202202006S).

## Author contributions

WL and SC designed the study. SC and CN conducted experiments. WL analyzed the data. WL and SC prepared and revised the manuscript. All the authors contributed to the article and approved the submitted version.

## Funding

This study was financially supported by grants from the National Natural Science Foundation of China (82001056) and Shanghai Sailing Program (21YF1439900).

## Conflict of interest

The authors declare that the research was conducted in the absence of any commercial or financial relationships that could be construed as a potential conflict of interest.

## Publisher’s note

All claims expressed in this article are solely those of the authors and do not necessarily represent those of their affiliated organizations, or those of the publisher, the editors and the reviewers. Any product that may be evaluated in this article, or claim that may be made by its manufacturer, is not guaranteed or endorsed by the publisher.

## Supplementary material

Supplementary Figure 1 Ranking of fungi species importance with *P. gingivalis* administration. Random forests algorithm was used to rank the importance of fungal species. The abscissa (Mean Decrease in Accuracy) is a measure of species importance, and the larger the value is, the more important the species is. The ordinate is the species in order of importance.

Supplementary Figure 2 Correlation of *Porphyromonadaceae an*d gut mycobiome at the family level. Correlation network analysis revealed strong positive- and negative-correlation associations between *Porphyromonadaceae* and fungal family taxa. Spearman correlation of > 0.6 or < - 0.6 between fungal family was represented.

Supplementary Figure 3 Association of serum metabolites and gut mycobiome. Heatmap of gut mycobiome and serum metabolites with Spearman correlation. Color denotes positive (red) and negative (blue) correlation values. **P* < 0.05, ***P* < 0.01, ****P* < 0.001.

Supplementary Table 1 Composition of gut fungi at species level both in *P. gingivalis* and control groups.
