# Supplementary figures and images for: Multi-omics insights reveal the remodeling of gut mycobiome with P. gingivalis
Source: Front Cell Infect Microbiol. 2022 Aug 29;12:937725. doi: 10.3389/fcimb.2022.937725 (PMC9465408; doi:10.3389/fcimb.2022.937725)

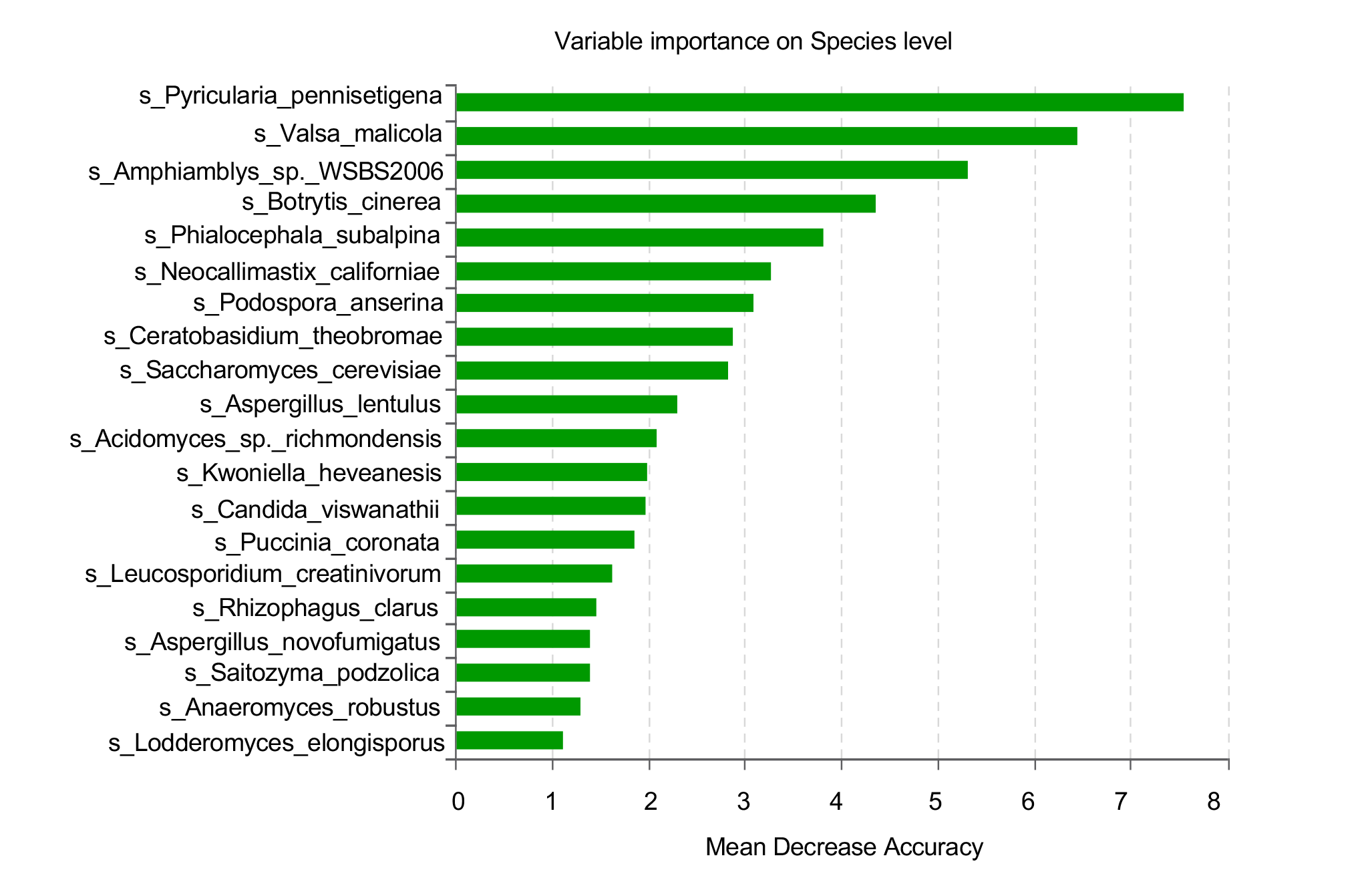

Supplement: Supplementary Figure 1 — Ranking of fungi species importance with P. gingivalis administration. Random forests algorithm was used to rank the importance of fungal species. The abscissa (Mean Decrease in Accuracy) is a measure of species importance, and the larger the value is, the more important the species is. The ordinate is the species in order of importance. [file Image_1.tif]

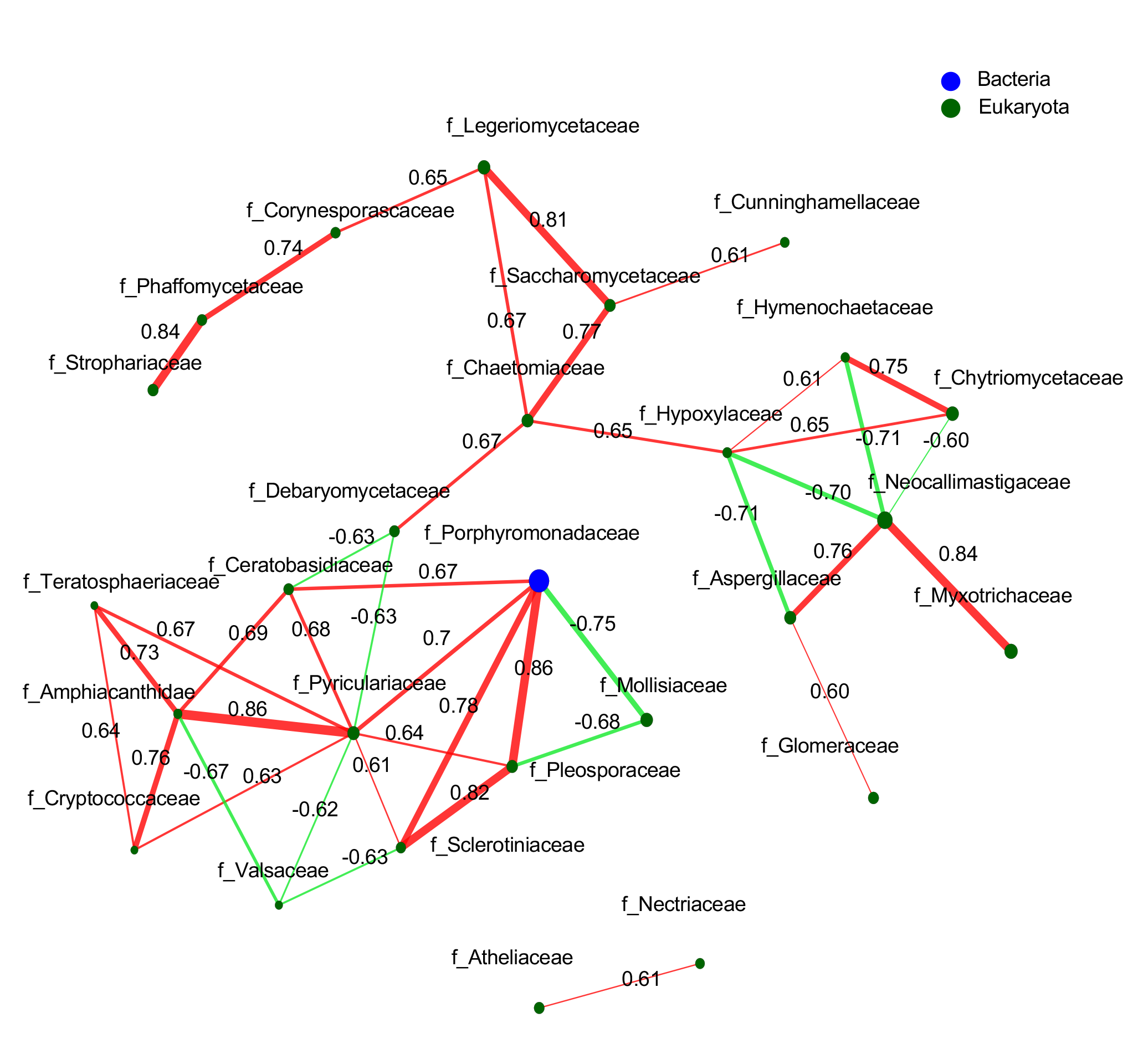

Supplement: Supplementary Figure 2 — Correlation of Porphyromonadaceae and gut mycobiome at the family level. Correlation network analysis revealed strong positive- and negative-correlation associations between Porphyromonadaceae and fungal family taxa. Spearman correlation of > 0.6 or < - 0.6 between fungal family was represented. [file Image_2.tif]

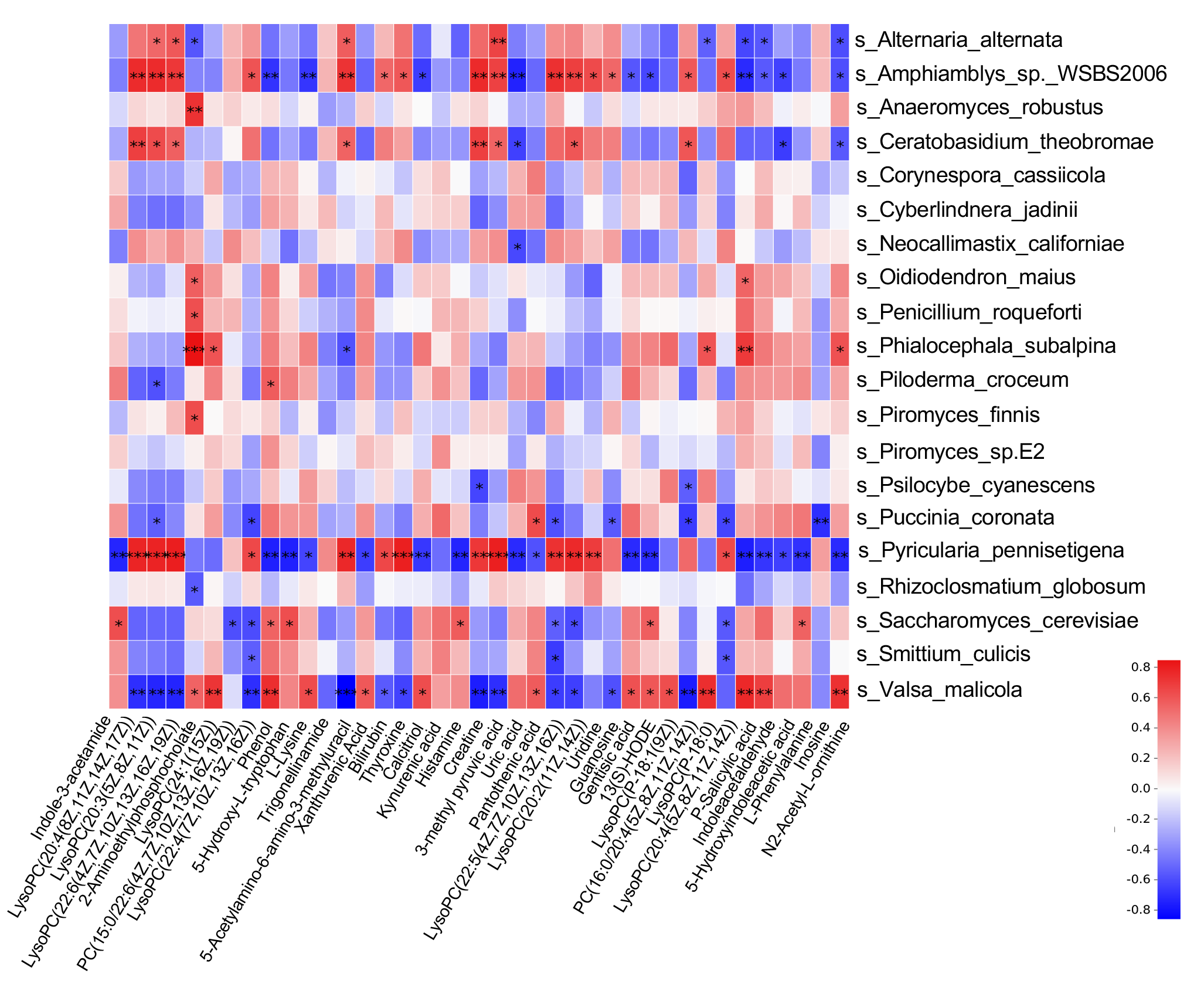

Supplement: Supplementary Figure 3 — Association of serum metabolites and gut mycobiome. Heatmap of gut mycobiome and serum metabolites with Spearman correlation. Color denotes positive (red) and negative (blue) correlation values. *P < 0.05, **P < 0.01, ***P < 0.001. [file Image_3.tif]
